# Changes in work situation and work ability in young female and male workers. A prospective cohort study

**DOI:** 10.1186/1471-2458-12-694

**Published:** 2012-08-24

**Authors:** Maria Boström, Judith K Sluiter, Mats Hagberg

**Affiliations:** 1 Occupational and Environmental Medicine, Department of Public Health and Community Medicine, University of Gothenburg, Gothenburg, Sweden; 2 Academic Medical Center, Department: Coronel Institute of Occupational Health, PO Box 22700, Amsterdam, 1100 DE, The Netherlands

**Keywords:** Work ability score, Work exposure, Epidemiology, Risk factors

## Abstract

**Background:**

Good work ability is very important in young workers, but knowledge of work situations that influence work ability in this group is poor. The aim of this study was to assess whether changes in self-reported work factors are associated with self-reported work ability among young female and male workers.

**Methods:**

A sample of 1,311 (718 women and 593 men) was selected from a Swedish cohort of workers aged 21–25 years. At baseline and at 1-year follow-up, participants completed a self-administrated questionnaire including ratings of physical and psychosocial work factors and current work ability. Prevalence ratios were calculated to assess univariate and multivariate associations between changes in work factors and changes in work ability.

**Results:**

Decreased job control (PR 1.7, 95% CI 1.49–2.12) and increased negative influence of job demands on private life (PR 1.5, 95% CI 1.25–1.69) were associated with reduced work ability for both female and male workers in the multivariate analyses. Among female workers, an association was found between improved work ability and increased social support at work (PR 2.4, CI 1.43–3.95). For male workers, increased job control (PR 2.3, 95% CI 1.21–4.54) and decreased negative influence of job demands on private life (PR 2.1, 95% CI 1.10–3.87) were associated with improved work ability in the multivariate analyses.

**Conclusions:**

Decreased job control and increased negative influence of job demands on private life over time seem to be the most important work factors associated with reduced work ability among young workers of both sexes. Increased social support at work, increased job control, and decreased negative influence of job demands on private life were also found to be the main work factors associated with improved work ability, although with possible gender differences.

## Background

Few studies have investigated work ability in young adults entering the job market. Despite research on adult working populations, the relationship between work factors and work ability is a considerably new area in the study of young working adults. It is very important for this group to sustain good work ability throughout their careers [1].

In occupational health, the concept of work ability refers to the balance between an individual’s resources such as health, functional capacity, knowledge, skills, attitudes, and motivation, and working conditions such as content, demands, and organizational supervisory management [1]. One often used self-report measure is the Work Ability Index (WAI) [2]. The work ability score (the first dimension of the WAI) has been used to rate general work ability in several studies [3-7].

Work-related factors are among the most important factors associated with work ability among adults [8]. Although associations and relationships between specific work factors and poor or good work ability among adults have been reported [9], few such studies have been longitudinal in their design.

Several work factors have been shown in cross-sectional research to associate with work ability in young adults, aged 18–29 years [10]. High physical work demands and mental strain at work were correlated with poor work ability, and appreciation at work, educational level, quality of life, and physical fitness were associated with excellent work ability, as measured on the WAI. However, few studies of work factors and work ability have been conducted in groups consisting entirely of young adults.

Despite data showing primarily cross-sectional associations between work and work ability in both younger and older adults, little is known about the effects of *changes* in work situations on work ability over time. Such knowledge could improve intervention strategies. A Finnish study with a follow-up of 11 years found relationships between increased muscular work, a greater number of difficult work postures, decreased opportunities for development and influence at the workplace, and reduced work ability in adult workers ≥ 44 years old [11]. The same study found relationships between decreased repetitive movements and increased satisfaction with the supervisor’s attitude, and improved work ability. Other studies conducted over 2–10 years have shown relationships between improved work ability and increased opportunities to influence one’s work, decreased mental and physical demands at work [12], and better job control and support [13]. These associations were found for groups of adult employees and managers with a mean age of 42 and 44 years respectively. For adults workers, low mental and physical work strain at midlife have been found to be related to the retention of work ability for a period of 28 years [3]. In all of these studies the WAI or the work ability score was used.

Although some studies include smaller groups of young workers [12-14], many of these results are for adult populations over 25 years of age. To our knowledge no studies of the possible causes of changes in work ability have been conducted exclusively among workers aged 20–25 years. Good work ability is of particular importance to this group, which has recently entered working life and will probably work for many years. There is also a lack of research into gender differences in factors related to changes in work ability. Such research, with separate analyses for the sexes, is important because young women and men still have different social roles, and may therefore, have or encounter different assumptions, opportunities, expectations in society and at work [15,16]. Continuous measurement of work ability and changes in work ability in a population of young working women and men, stratified by gender, is therefore greatly needed. Such knowledge would provide a good base for interventions preventing the deterioration, and promoting the maintenance and improvement, of work ability in young employees of both sexes.

The aim of this study was to assess whether a change in self-reported work factors over time was associated with self-reported work ability among young workers. Two research questions were formulated: i) Which changes in work factors were associated with reduced or improved work ability? and ii) Were these changes in work factors similarly applicable to both female and male workers?

## Methods

### Study design and cohort

Data for this study was obtained from a prospective population study of young adults in Sweden, aged 20–24 years, with a 1-year follow-up. The baseline cohort of 7,125 individuals, which overrepresented women and those of Swedish birth, was the basis of a project called Work Ability of Young Adults (WAYA). This cohort was derived from a questionnaire sent to an equal number of women and men in a randomly selected sample of 20,000 young adults. The aim of this project was to follow young adults over time with questionnaires focusing on contemporary exposures to work factors such as information and communication technology (ICT), environmental factors related to lifestyle, exposures at work or during studies, health, productivity, and work ability [17]. The 1-year follow-up cohort consisted of 4,163 individuals.

### Study sample

**Figure 1:**
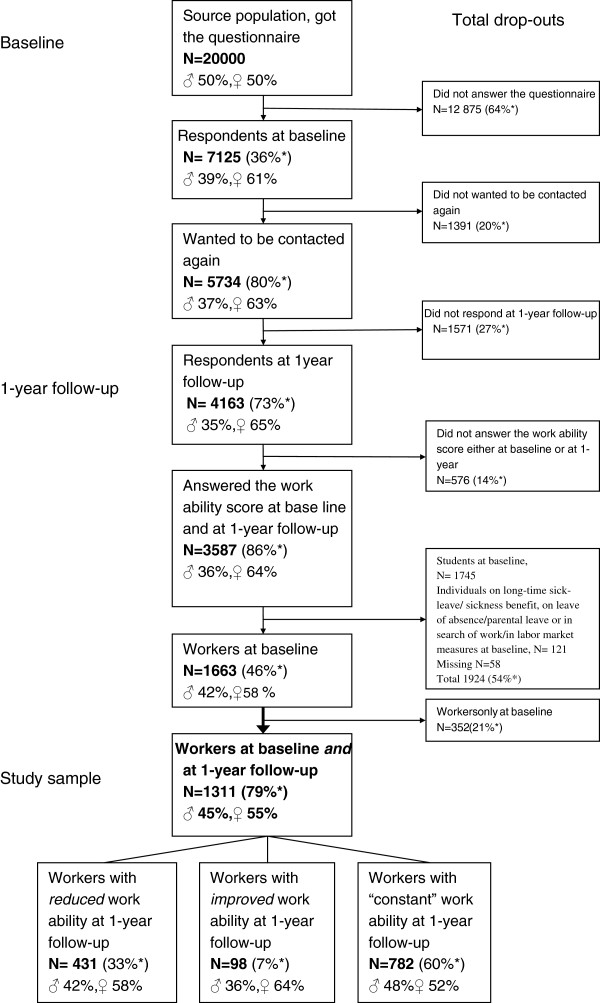
**Selection of the study sample. **The study sample, including workers who had answered the work ability score twice and had reported salaried work at both measurements, was obtained from the 1 year follow-up cohort.

The study sample consisted of 1,311 young adults, aged 21–25 years, here defined as young workers, with adult workers defined as those > 25 years. The inclusion criteria for this group from the 1-year follow-up cohort, were i) having answered the work ability score (the first dimension of the WAI) at both baseline and follow-up, and ii) having salaried work at both baseline and the 1-year follow-up. This resulted in the exclusion of 1,745 students, (Figure 1).

### Drop-outs

The drop-out group of workers (not including students) consisted of 1,500 individuals (not shown in the figure). Of these, 1,490 workers did not answer the follow-up questionnaire. Another 10 individuals answered the follow-up questionnaire, but did not answer the work ability score.

A drop-out analysis showed that the lost group had similar scores to the study sample for several work factors at baseline, such as job control, social support at work, reward relative to effort, and negative influence of job demands on private life. However, the drop-out group consisted of significantly more men, (a 5% difference) than the study sample. Also, the workers in the drop-out group had a significantly lower daily use of the computer in general (a 7% difference) than workers in the study sample. For work ability, a statistically significant small difference (0.1 score levels) could be seen between the groups, but this is most likely not of clinical interest.

### Data selection

Data was collected in 2007 and 2008 through two self-administrated questionnaires consisting of 78 items. The first questionnaire was sent by post and the second by web, after a one-year interval. As compensation, invited participants received a lottery ticket valued at 1 Euro with each questionnaire. The posting of the first questionnaire was followed by two reminders, and the second by three, the last including two cinema tickets. The response at baseline was 36%, and at the 1-year follow-up, 73%. This procedure has been described in detail [18].

Descriptive data were collected from the study sample at baseline, and work factors and self-reported level of work ability were collected through the questionnaires at both baseline and the 1-year follow-up needed for the analyses.

### Individual characteristics

Questions about descriptive data were partly selected from previously shown associations and relationships between work ability and individual factors for both adults [9] and young adults [10]. Consequently, individual factors such as sex, civil status, educational level, main occupation, living area, country of birth, and health-related questions about smoking, body mass index (BMI), physical activity, chronic pain, symptoms of depression, and experienced health, were used.

### The outcome

#### Work ability

The WAI is a self-report instrument consisting of seven dimensions derived from ten items, on which individuals estimate the dimensions of their own work ability [2]. This instrument has previously been used for workers as young as 16 years of age [7,14]. The WAI has been shown to be a useful tool when investigating an entire working population, although further evaluation of the instrument is needed for workers of different ages [19].

#### The change in self-reported work ability

Work ability in this study was measured by the work ability score, an “age-free” item according to Ilmarinen [1]. This one item of work ability measures “current work ability compared with the lifetime best” and consists of a scale from 0 representing “cannot work at all right now” to 10 representing “my work ability is at its best right now”. A change in the work ability score has been validated to show a change in the entire WAI for women on long-term sick-leave [20].

We defined a real change in the work ability score as a decrease or increase of 2 score levels or more, based on prior analyses in a test/retest study of 29 young adults [17], in which the smallest detectable change was calculated as 1.9. Self-reported changes in work ability, in any direction, were dichotomized to 1 for changes of 2 score levels or more, and to 0 for changes of 1 score level or none.

### The explanatory variable

#### Physical factors at work

Because of a lack of knowledge about how changes in work factors influence work ability in young workers, the selection of physical work factors was based on work factors previously found to be associated with changes in work ability in a mainly adult working population [11-13].

The time frame for all questions, except on vibration exposure during the last year, was the last 30 days.

Two questions addressed computer use. The first question asked about total daily time spent at a computer, for both work and leisure. Possible response alternatives were < 2 h/day, 2–4 h/day, and > 4 h/day. The second question concerned computer use of more than 2 hours with no breaks longer than 10 minutes, with possible answers as never, once in a while, a couple of times per month, a couple of times per week, and most days. The cut-off points for these questions were obtained from a cross-sectional study [21].

Questions about work postures had different response alternatives. “How long daily do you work with your hands above shoulder level,” could be answered by never, < 1 hour, 1–2 hours, and > 2 hours [22]. Similar responses to the question, “How long each day do you work with a flexed or extended neck” could be never, < 3 hours, 3–5 hours, and > 5 hours [23]. “How long daily do you work with a flexed back” could be answered by never, < 0.5 hour, 0.5–1 hour, and > 1 hour [24].

Questions about lifting had the answer alternatives for intensity of 5–10 kg, 11–15 kg, 16–25 kg, and > 25 kg and for frequency of 0–4, 5–15, 16–30, and > 30 times/day [25]. A question about the frequency with which they handled tools or equipment demanding a forceful grip to the equivalent of lifting 1 kg or more had the alternatives of seldom or never, several times per day, several times per hour, and several times per minute.

For the question concerning regular use of vibrating hand-held machines at work, the alternatives were yes or no.

#### Psychosocial factors at work

Like the physical work factors, psychosocial work factors were selected from known relationships between changes in psychosocial work factors and work ability reported mainly in adult workers [11-13].

Questions related to job demands, job control, social support at work, and reward relative to effort had the same response alternatives: corresponds very poorly, corresponds somewhat poorly, corresponds fairly well, and corresponds very well.

Job demands were defined as exposure to high demands and expectations at work, and job control as having control over and the ability to deal with situations at work. Questions about social support concerned access to support and help at the workplace in the event of problems. These one-item questions were developed from the demand–control model [26], which also included social support from co-workers in a later version [27].

From the effort–reward model [28], one-question, concerning the reward deserved in relation to the effort extended and the actual production at work was modified. There was also a question about whether demands at work negatively influenced private life (leisure, home, and family life), with the possible response alternatives very seldom, fairly seldom, sometimes, fairly often, and very often. This question, derived from the model of work–home interference [29], has been validated [30].

Two new questions, asking about the previous month, were constructed to address flexibility in work [31], with the same response categories: never, once in a while, a few times per month, a few times per week, or more or less daily. The first question asked whether work was performed outside the workplace, for example at home, and the second, whether respondents had ever to be available by mobile phone after working hours. One further question asked how often they had been working more than 12 hours in a day within the last 30 days. Possible responses were 0, 1–2, 3–8, 9–15, and > 15 times in the last month.

A question about whether they experienced noise annoyance at the workplace [32] could be answered by never, once in a while, a few times per month, a few times per week, or more or less every day.

#### Changes in self-reported work factors

A changed answer for any of the work factors by one step or more among the three to five response alternatives between baseline and the 1-year follow-up was defined as a change. This simplified measure of change was chosen despite diverse methods of interpreting scales [33], based partly on the test/rest study [17] in which the numbers of response alternatives were changed for some questions to increase reliability.

### Statistical analyses

For all analyses in this study, SAS version 9.1 (SAS Institute, Cary, NC) was used.

Descriptive data of the sample and subgroups at baseline were first derived through frequency analyses.

Prospective analyses were performed next to assess associations between changes in work factors and changes in work ability. The Cox proportional hazard regression model was used to estimate prevalence ratios (PR) in both univariate and multivariate analyses, with time set to 1 [34]. These analyses were carried out for the sample as a whole [n = 1,311] adjusted for sex, and also stratified by gender as recommended [35]. For a more correct confident interval (CI)(95% CI) the robust variance was used [34]. In this study, prevalence refers to the proportion of individuals reporting reduced or improved work ability.

In the analyses of reduced work ability, the reference group consisted of those with either constant work ability or improved work ability at the 1-year follow-up (n = 880). The reference group in the analyses for improved work ability consisted of those with either constant work ability or reduced work ability at the 1-year follow-up (n = 1,213).

The work factor variables were coded so that a PR > 1 for reduced work ability meant that an increase in a work factor was hypothesized to have a negative effect on work ability. For improved work ability, a PR > 1 meant that an increase in a work factor was hypothesized to have a positive effect on work ability.

Finally, before the backward stepwise multivariate regression analysis, work factors with a *p*-value ≤ 0.2 in the univariate Cox proportional hazard regression analysis were selected for further analysis. Next, Spearman’s rank correlation was calculated amongst these selected work factors to check for multicollinearity. All paired correlations were < 0.8 and hence no multicollinearity was found. In the backward stepwise multivariate regression analysis, the variables with the highest *p*-values were excluded one at a time in order. When all variables had a *p*-value ≤ 0.05 the step-wise procedure was finished.

The study required no approval from the Regional Ethics Review Board in Gothenburg.

## Results

### Characteristics of the study sample at baseline

The prevalence in the study sample of reduced work ability was 33% and of improved work ability was 7%. The young workers had high level of self-reported work ability (Table 1), compared to self-reported health, which was also seen in employed Finnish youth [10]. The study sample (N = 1,311) consisted of slightly more women than men. Nearly one fifth of the individuals had finished college or university. However, only 11% of the individuals had occupations that required their higher level of education. These occupations included teachers, nurses, engineers and economists. The largest proportion of workers, nearly nine out of ten, reported occupations that did not demand a higher education, such as nurses assistant, fitter, postman, personal assistant, salesman, shop assistant, secretary, construction worker, and carpenter. There was no overall similarity between women and men for several categories of the descriptive data.

**Table 1:** Characteristics of the study sample at baseline

	**Males**		**Females**	
	**(N = 593, 45%)**		**(N = 718, 55%)**	
**Work ability**; mean, (range), SD	8.7 (1–10) 1.3		8.5 (1–10) 1.6	
**Individual factors at baseline**				
	N	%	N	%
**Civil status**				
Cohabit/married/partnership	197	35	308	47
Girl-/boyfriend, not living together	115	20	139	21
Single	252	45	210	32
**Education level – highest finished**				
Compulsory school/high school	516	83	540	73
College/university	72	17	168	27
**Occupation with demands of education at college/university level**				
Yes	44	8	102	15
No	538	92	594	85
**Living area**				
City	222	37	310	43
Not city	371	63	408	57
**Birth country**				
Sweden	559	94	678	94
Other	34	6	40	6
**Smoking**				
No, not at all/seldom	529	89	594	83
Yes, daily/nearly daily	64	11	123	17
**Body mass index**				
< 25 kg/m^2^	372	64	511	76
≥ 25 kg/m^2^	206	36	161	24
**Physical activity in leisure time**				
Moderate exercise-hard training	487	83	601	85
Sedentary	99	17	106	15
**Chronic pain or ache ( >3 months)**				
No	487	83	501	70
Yes	99	17	215	30
**Symptoms of depression last month**				
No	301	69	249	50
Yes	138	31	250	50
**Experienced health**				
Good or very good	491	83	517	72
Very bad, bad or moderately	102	17	198	28

N = 1,311. (*SD* = standard deviation, *N* = number of workers).

The subgroups consisted of individuals with reduced work ability (n = 431), improved work ability (n = 98), and constant work ability (n = 782) at the 1-year follow-up (Additional file 1: Table A). As a group, workers with improved work ability at the follow-up had had the lowest level of work ability at baseline, 6.1, of all subgroups and they reported poorer health than the other groups.

The study sample reported varying exposures to both physical and psychosocial work factors (Additional file 2: Table B). No major gender differences were found in this report, although male workers seem to report higher exposure to lifting. Overall changes from baseline to follow-up ranged from 4% of the workers reported increased and decreased use of vibrating tools to 33% of the workers reported increased noise annoyance at the workplace.

### Associations between changes in work factors and work ability

Decreased job control, social support at work, reward relative to effort, and increased negative influence of job demands on private life, were associated with reduced work ability, as seen in the univariate analyses for the sample both as a whole and stratified for female and male workers (Table 2). For female workers, increased work outside the workplace was also associated with reduced work ability in these analyses. In total, 4 work factors were statistically significant in relation to change in work ability for the sample; the remaining 16 work factors had PRs between 0.9–1.2, and were not statistically significant in the univariate analyses.

In the multivariate analyses, decreased job control and increased negative influence of job demands on private life were shown to be the most important work factors associated with reduced work ability for both female and male workers.

**Table 2:** Prospective relationships between changes in work factors and reduced work ability

	**All workers N = 1,311**						**Males N = 593 (45%)**						**Females N = 718 (55%)**					
			**Univariate analysis**^**1**^		**Multivariate analysis**^**2**^				**Univariate analysis**		**Multivariate analysis**^**3**^				**Univariate analysis**		**Multivariate analysis**^**3**^	
	**Exposed**	**Cases**	**PR**	**95% CI**	**PR**	**95% CI**	**Exposed**	**Cases**	**PR**	**95% CI**	**PR**	**95% CI**	**Exposed**	**Cases**	**PR**	**95% CI**	**PR**	**95% CI**
**Changes in physical work factors**																		
**Increased daily computer use in general**	239	82	1.0	0.86-1.28			102	32	1.0	0.75-1.42			137	50	1.1	0.83-1.36		
**Decreased rests during computer use in general**	378	124	1.0	0.84-1.18			158	50	1.1	0.80-1.38			220	74	1.0	0.76-1.19		
**Increased time with hands above shoulder level**	246	77	0.9	0.77-1.15			108	32	1.0	0.70-1.33			138	45	0.9	0.71-1.20		
**Increased time with flexed or extended neck**	345	112	1.0	0.82-1-17			154	50	1.1	0.83-1.42			191	62	0.9	0.72-1.15		
**Increased time with flexed back**	297	94	1.0	0.79-1.16			142	43	1.0	0.74-1.32			155	51	0.9	0.72-1.20		
**Increased lifting 5–10 kg**	249	77	0.9	0.76-1.15			127	32	0.8	0.57-1.09			122	45	1.1	0.83-1.39		
**Increased lifting 11–15 kg**	189	64	1.1	0.85-1.32			112	37	1.1	0.82-1.48			77	27	1.0	0.73-1.39		
**Increased lifting 16–25 kg**	136	46	1.1	0.82-1.36			84	25	1.0	0.68-1.38			52	21	1.2	0.83-1.66		
**Increased lifting > 25 kg**	115	42	1.2	0.89-1.48			70	23	1.1	0.76-1.56			45	19	1.2	0.86-1.76		
**Increased forceful grip**	221	82	1.2 ^a^	0.97-1.43			119	42	1.2	0.91-1.59			102	40	1.2	0.88-1.50		
**Increased use of vibrating tools**	58	21	1.1	0.79-1.60			33	11	1.1	0.67-1.81			25	10	1.2	0.71-1.89		
**Changes in psychosocial work factors**																		
**Increased job demands**	385	137	1.1	0.94-1.31			150	47	1.0	0.79-1.36			235	90	1.2	0.94-1.42		
**Decreased job control**	388	187	**1.8 ^a^**	**1.57-2.11**	**1.7**	**1.49-2.12**	168	78	**1.9 ^a^**	**1.52-2.42**	**1.7**	**1.36-2.23**	220	109	**1.8 ^a^**	**1.44-2.12**	**1.6**	**1.34-1.99**
**Decreased social support at work**	420	173	**1.4 ^a^**	**1.21-1.65**	**1.2**	**1.00-1.38**	179	75	**1.6 ^a^**	**1.29-2.08**	**1.4**	**1.06-1.74**	241	98	**1.3 ^a^**	**1.04-1.56**		
**Decreased reward relative to effort**	415	159	**1.3 ^a^**	**1.08-1.48**			189	69	**1.3 ^a^**	**1.03-1.68**			226	90	1.2 ^a^	0.99-1.50		
**Increased negative influence of job demands on private life**	411	180	**1.6 ^a^**	**1.34-1.82**	**1.5**	**1.25-1.69**	172	71	**1.6 ^a^**	**1.24-2.01**	**1.5**	**1.20-1.91**	239	109	**1.6 ^a^**	**1.27-1.89**	**1.4**	**1.15-1.71**
**Increased work outside the workplace**	317	117	1.2 ^a^	0.98-1.38			138	43	1.0	0.77-1.37			179	74	**1.3 ^a^**	**1.02-1.57**		
**Increased overtime work (>12 h/day)**	319	109	1.1	0.89-1.27			167	54	1.1	0.83-1.41			152	55	1.0	0.83-1.34		
**Increased reachable by mobile phone out of work time**	340	122	1.1	0.96-1.35			172	61	1.2 ^a^	0.97-1.60			168	61	1.1	0.84-1.33		
**Increased noise annoyance at the workplace**	427	154	1.1 ^a^	0.98-1.35			190	60	1.1	0.81-1.36			237	94	1.2 ^a^	0.99-1.50		

* The figures in boldface were statistically significant (the lower limit of the 95% CI being > 1.00).

^1^ Adjusted for sex.

^2^ Backward stepwise analyses, adjusted for sex.

^3^ Backward stepwise analyses.

^a^ Work factor with a p-value ≤ 0.2 in the univariate analysis and accordingly included in the starting multivariate model.

The results are presented for the study sample in whole and for female and male workers separated*.

(*N* = number of workers, *cases* = exposed individuals reported reduced work ability, *PR* = prevalence ratios, *95% CI* = 95% confidence interval).

Increased social support at work, job control, and reward relative to effort, and decreased negative influence of job demands on private life were associated in the univariate analyses with improved work ability (Table 3). Furthermore, decreased time with hands above shoulder level and decreased daily computer time in general showed similar associations. In total, 6 work factors were statistically significant in these analyses, 13 work factors had PRs between 0.9–1.2 and were not statistically significant, and 1 work factor had only 3 cases.

In the multivariate analyses, increased social support at work, increased job control, decreased daily computer use in general, and decreased negative influence of job demands on private life were the most important factors associated with improved work ability. Possible gender differences were seen between female and male workers.

**Table 3:** Prospective relationships between changes in work factors and improved work ability

	**All workers N = 1,311**						**Males N = 593 (45%)**						**Females N = 718 (55%)**					
			**Univariate analysis**^**1**^		**Multivariate analysis**^**2**^				**Univariate analysis**		**Multivariate analysis**^**3**^				**Univariate analysis**		**Multivariate analysis**^**3**^	
	**Exposed**	**Cases**	**PR**	**95% CI**	**PR**	**95% CI**	**Exposed**	**Cases**	**PR**	**95% CI**	**PR**	**95% CI**	**Exposed**	**Cases**	**PR**	**95% CI**	**PR**	**95% CI**
**Changes in physical work factors**																		
**Decreased daily computer use in general**	201	23	**1.7 ^a^**	**1.10-2.65**	**1.8**	**1.15-2.76**	94	9	1.8 **^a^**	0.89-3.80			107	14	1.6 **^a^**	0.93-2.85	1.7	0.99-3.00
**Increased rests during computer use in general**	364	30	1.2	0.76-1.74			169	10	1.0	0.49-2.04			195	20	1.2	0.75-2.07		
**Decreased time with hands above shoulder level**	273	30	**1.7 ^a^**	**1.13-2.55**			129	12	1.9 **^a^**	0.96-3.67			144	18	1.6 **^a^**	0.95-2.67		
**Decreased time with flexed or extended neck**	309	26	1.2	0.76-1.80			142	9	1.1	0.53-2.29			167	17	1.2	0.72-2.07		
**Decreased time with flexed back**	293	25	1.2	0.78-1.86			140	10	1.3	0.64-2.63			153	15	1.2	0.66-2.00		
**Decreased lifting 5–10 kg**	270	21	1.0	0.66-1.67			120	9	1.4 **^a^**	0.66-2.84			150	12	0.9	0.49-1.63		
**Decreased lifting 11–15 kg**	223	16	1.0	0.61-1.70			132	11	1.6 **^a^**	0.80-3.18			91	5	0.6	0.24-1.44		
**Decreased lifting 16–25 kg**	166	13	1.1	0.64-1.94			95	9	1.8	0.88-3.75			71	4	0.6	0.23-1.65		
**Decreased lifting > 25 kg**	131	11	1.2	0.67-2.24			81	7	1.6	0.71-3.50			50	4	0.9	0.34-2.39		
**Decreased forceful grip**	267	23	1.2	0.78-1.91			131	8	1.0	0.49-2.25			136	15	1.3	0.77-2.32		
**Decreased use of vibrating tools**	46	3	1.0	0.31-3.00			31	2	1.1	0.28-4.37			15	1	0.8	0.11-5.09		
**Changes in psychosocial work factors**																		
**Decreased job demands**	236	18	1.0	0.63-1.68			107	10	1.8 **^a^**	0.90-3.67			129	8	0.7	0.32-1.36		
**Increased job control**	199	29	**2.3 ^a^**	**1.56-3.52**	**1.8**	**1.18-2.83**	90	11	**2.6 ^a^**	**1.30-5.04**	**2.3**	**1.21-4.54**	109	18	**2.2 ^a^**	**1.35-3.71**		
**Increased social support at work**	239	35	**2.5 ^a^**	**1.68-3.63**	**2.0**	**1.34-3.05**	103	10	1.9 **^a^**	0.94-3.84			136	25	**2.8 ^a^**	**1.76-4.50**	**2.4**	**1.43-3.95**
**Increased reward relative to effort**	326	39	**2.0 ^a^**	**1.33-2.87**			133	11	1.6 **^a^**	0.80-3.15			193	28	**2.2 ^a^**	**1.36-3.48**	**1.7**	**1.03-2.82**
**Decreased negative influence of job demands on private life**	349	41	**2.0 ^a^**	**1.34-2.87**	**1.7**	**1.13-2.43**	149	15	**2.2 ^a^**	**1.18-4.25**	**2.1**	**1.10-3.87**	200	26	**1.8 ^a^**	**1.13-2.92**		
**Decreased work outside the workplace**	209	14	0.9	0.51-1.52			95	4	0.7	0.24-1.87			114	10	1.0	0.52-1.91		
**Decreased overtime work(>12 h/day)**	264	20	1.0	0.64-1.64			123	10	1.5	0.76-3.10			141	10	0.8	0.40-1.48		
**Decreased reachable by mobile phone out of work time**	249	21	1.2	0.74-1.86			117	7	1.0	0.46-2.27			132	14	1.3	0.72-2.23		
**Decreased noise annoyance at the workplace**	329	25	1.0	0.66-1.58			147	8	0.9	0.42-1.94			182	17	1.1	0.64-1.85		

* The figures in boldface were statistically significant (the lower limit of the 95% CI being > 1.00).

^1^ Adjusted for sex.

^2^ Backward stepwise analyses, adjusted for sex.

^3^ Backward stepwise analyses.

^a^ Work factor with a p-value ≤ 0.2 in the univariate analyses and accordingly included in the starting mutivariate model.

The results are presented for the study sample in whole and for female and male workers separated*.

(*N* = number of workers, *cases* = exposed individuals reported improved work ability *PR* = prevalence ratios, *95% CI* = 95% confidence interval).

## Discussion

Changes in job control and negative influence of job demands on private life seem to both reduce and improve work ability, although with possible gender differences in improved work ability. Increased social support at work also appears to improve work ability, mostly for young female workers.

### Reduced work ability associated with changes in work factors among young workers

Decreased job control in this study sample was one of the most important work factors associated with reduced work ability. This is in accordance with previous research in terms of decreased influence at work [11], a minimum level of control related to constant low work ability [13], and poor opportunities to control one’s own work [14], especially among adult workers, but even among those 19–25 years of age. The definition of job control in the present study is a feeling of control and ability to handle work situations, developed from the model of Karasek and Theorell [26]. However, their model has two dimensions for job control: skill discretion and decision authority. Consequently, slightly different measurements than those in the original model have been used, making a direct comparison with their results uncertain.

Decreased job control associated with reduced work ability for both women and men, has not, to our knowledge, been previously demonstrated in any study conducted exclusively in young workers. No major gender differences were found in our study, in accordance with Nordander [36], who showed the same experience of job control in both female and male adult workers. Moreover, the importance of job control for adult workers with reduced work ability to remain productive has been recently emphasized [37]. Job control therefore appears to be very important to workers independent of age and gender.

Increased negative influence of job demands on private life was also found to be associated with reduced work ability for both sexes in this study sample. Work–home interference is defined as situations in which negative or positive stress reactions at work influence a person’s function at home; this is more common than home–work interference in which stresses of home influence performance at work [38]. Young workers, both women and men, may have a greater wish or need than older workers to separate their work and their private lives, possibly because this age group may value individualism more highly.

### Improved work ability associated with changes in work factors among young workers

Increased social support at work was found to be one of the strongest work factors associated with improved work ability, as previously reported in terms of support from supervisors [12]. A high level of organizational support has also been shown to have a relationship to excellent work ability [13]. These results have been established in studies that included some young, but primarily adult, workers.

In our study, as shown in the multivariate analyses, increased social support at work seemed to have a stronger association with improved work ability in young female workers than in young male workers. One explanation could be that in this study sample, female and male workers had the traditional occupations often seen in the gendered labour market. Young women worked mainly, and more often than young men, with people. This type of work may require more social support than work in the traditional male sector.

Increased job control was also shown to be a potentially important work factor for improved work ability, as also seen in studies, mainly among adults, of increased influence [12]. Improvements in job control have previously been suggested to prevent reduced work ability, chiefly among adults [13]. Better job control in our study was seen as a possibly stronger work factor for men than for women, again perhaps due to the gendered labour market, in which predominantly male occupations may require a greater degree of individual control.

Decreased daily computer use was one of the few physical work factors found to be associated with improved work ability; a finding to our knowledge not previously shown for either adult workers or young workers. Computer time in leisure and computer mouse use at work, however, has both been shown to be related to reduced productivity [39,40].

Reducing the negative influence of job demands on private life also seems to be important for improved work ability. A better balance in work–home interference seems to improve young workers’ work ability, just as more work–home interference seems to be associated with reduced work ability. Male workers in particular might obtain better improvements in work ability from reduced work–home interference than from improvements in other work factors.

### Associations of work ability with changes in work in young workers as compared to adult workers

Changes in psychosocial work factors, as shown earlier in studies of predominantly adult workers [12-14], seem also to be important influences on work ability in young workers. In contrast to some of those results [12,14], however, changes in physical work factors in this group of solely young workers seem to be of less importance. Decreased daily computer use in general was the only physical work factor found to be associated with improved work ability. Increased physically strenuous work may not yet reduce younger workers’ work ability, but it may affect their health, as seen in the levels of reported chronic pain in this sample.

### Methodological considerations

Possible methodological limitations include causal interpretation, generalization, recall bias, the use a measurement primarily used with adults, a potential regression towards the mean, and ceiling effects. The strengths of this study, however, include its prospective design, the large study group, and its contribution to this relatively unexamined research area of work ability in young workers. Despite the power of the prospective design, the results should be carefully interpreted as prospective findings only, since changes in work ability and changes in work factors are measured simultaneously at the 1-year follow-up. However, the study design, with its aim to assess changes, contributes more to the research in this field than would a cross-sectional study with only baseline results.

The results of this study could be generalized to a group of young workers in occupations not demanding a higher education. However, as Swedish-born youths were overrepresented in the baseline cohort, it is not clear whether the results could be generalized to foreign-born workers. Because the drop-out group of non-student workers consisted of more men than women and had less daily computer use in general than the remaining sample, attention to the possibility of a selective study sample bias is important. Nevertheless, because we included gender stratification in the analysis, the difference in sex distribution is probably not a problem. The association between decreased daily computer use in general and improved work ability is not emphasized, due to a possible ceiling effect.

Recall bias is common in survey-based studies [41]. To prevent this in the current study, a first questionnaire was used in a pilot study among 36 young adults, 16–22 years old. A test-retest was then performed in another group of 31 young adults and used to modify several questions. (Neither of these studies has been published).

The use of the Work Ability index (WAI) with its work ability score is considered to be a useful method for this study sample. Even if young workers do not yet have the experience with which to compare their work ability with their life-time best, the work ability score in the WAI has been recommended for use in younger workers [1]. Defined change in work ability was calculated from the work ability score of the WAI, [11].

The interpretation of changes in work factors contributing to improved work ability should be performed with caution. Because the subgroup that reported better work ability at the 1-year follow-up had begun with lower work ability and poorer health at baseline (Additional file 1: Table A) than the other subgroups, a regression to the mean cannot be excluded. However, separate post-hoc analyses to account for this phenomenon did not change the associations between improved work ability and changes in work.

Regression to the mean could also make the interpretation of associations between reduced work ability and changes in work difficult. As separate post-hoc analyses showed no differences in the main results between two groups with different baseline levels of work ability, the conclusions remain unchanged.

The possible ceiling effect for improved work ability was assessed with separate post-hoc analyses that showed, in contrast to earlier results, no significance for decreased daily computer use in general associated with improved work ability. The interpretation of this result was unsure and consequently not included in the conclusions of this study.

No correlations for confounders were calculated. A change in pain was discussed as a possible confounder. However, it has been shown that people with musculoskeletal disorders do not over-report their exposure [42]. Further, little influence on reported job strain, in terms of negative affectivity, has been shown for depressive symptoms [43].

Accordingly, despite some limitations and weaknesses, the current study may contribute to new knowledge and new tools for interventions to maintain or improve work ability among young workers.

### Applications

Several researchers have emphasized that work ability is a continuum [44] and that two aspects are needed for work interventions: the prevention of poor work ability and the promotion of excellent work ability [13]. Based on findings from the current study, there are many ways to promote good work ability and prevent loss of work ability, mainly through improvements in psychosocial work factors. Changes in job control and in the negative influence of job demands on private life are the factors that most affect young workers. Supporting work ability primarily through improving those psychosocial conditions seems feasible, perhaps using somewhat different approaches for young women and young men.

The suggestions related to the findings of the current study are in partial agreement with previous recommendations for mainly adult workers [12-14]. However, work ability among young workers may also depend on that group’s interpretation and idea of work [10], which could make prevention and promotion more complex. Consequently, more research is needed in this area. Furthermore, in contrast to intervention suggestions aimed mainly towards improving the capacity and performance of workers [45], this study proposes the importance of intervention strategies aimed towards influencing work situations that may affect workers’ ability.

## Conclusions

Decreased job control and increased negative influence of job demands on private life over time seem to be the most important work factors associated with reduced work ability among young female and male workers. Increased social support at work, increased job control, and decreased negative influence of job demands on private life, with possible gender differences, were found to be the main work factors associated with improved work ability in young workers.

## Competing interests

The authors declare that they have no competing interests.

## Authors’ contributions

MB, MH and JS designed the study. MB performed the data analysis, MH supervised the analysis, and MH and JS contributed to the interpretation of the data. MB wrote the manuscript and MH and JS discussed the manuscript with MB and made a contribution to its final form. All readers have read and approved the manuscript.

## Pre-publication history

The pre-publication history for this paper can be accessed here:

http://www.biomedcentral.com/1471-2458/12/694/prepub

## Supplementary Material

Additional file 1 **Table A. **Descriptive characteristics of the subgroups of the study sample at baseline. (SD = standard deviation, N = number of workers). Description of the data: The table gives the reader a view of the subgroups in the study sample at baseline concerning background data, as civil status, educational level, different health factors, BMI, physical activity etc. Click here for file

Additional file 2 **Table B. **Descriptive characteristics of work factors at baseline and changes in work factors between baseline and 1- year follow-up for the study sample.(N = number of workers). Description of the data: this table gives a completion to the manuscript and show the exposure at baseline and the change in exposure between baseline – the 1-year follow-up, to better assess the results and the discussion in the article. Click here for file
