# Supplementary material for: Changes in work situation and work ability in young female and male workers. A prospective cohort study
Source: BMC Public Health. 2012 Aug 24;12:694. doi: 10.1186/1471-2458-12-694 (PMC3508794; doi:10.1186/1471-2458-12-694)
Supplement: Additional file 1 — Table A. Descriptive characteristics of the subgroups of the study sample at baseline. (SD = standard deviation, N = number of workers). Description of the data: The table gives the reader a view of the subgroups in the study sample at baseline concerning background data, as civil status, educational level, different health factors, BMI, physical activity etc. [file 1471-2458-12-694-S1.doc]

| **Table A. Descriptive characteristics of the subgroups of the study sample at baseline. (SD= standard deviation, N=number of workers)** | The subgroup of workers with **reduced** work ability at the 1- year follow-up  N= 431 | | Males (N=181, 42%) | | Females  (N=250, 58%) | | The subgroup of workers with **improved** work ability at the 1- year follow-up  N= 98 | | Males (N=35, 36%) | | Females (N=63, 64%) | | The subgroup of workers with **constant** work ability at the 1-year follow-up  N= 782 | | Males  (N=377, 48%) | | Females (N=405, 52%) | |
| --- | --- | --- | --- | --- | --- | --- | --- | --- | --- | --- | --- | --- | --- | --- | --- | --- | --- | --- |
|  |  |  |  |  |  |  |  |  |  |  |  |  |  |  |  |  |  |  |
| Work ability at baseline, mean (range), SD | 9.2 (5-10) 1.0 | | 9.3 (7-10) 0.9 | | 9.1 (5-10) 1.1 | | 6.1 (1-8) 1.7 | | 6.5 (1-8) 1.8 | | 5.8 (1-8) 1.8 | | 8.6 (3-10) 1.3 | | 8.7 (4-10) 1.3 | | 8.5 (3-10) 1.3 | |
| *Individual factors at baseline* |  |  |  |  |  |  |  |  |  |  |  |  |  |  |  |  |  |  |
|  | N | % | N | % | N | % | N | % | N | % | N | % | N | % | N | % | N | % |
| Civil status   Cohabit/married/partnership |  |  |  |  |  |  |  |  |  |  |  |  |  |  |  |  |  |  |
|  | 165 | 42 | 59 | 35 | 106 | 47 | 33 | 36 | 11 | 31 | 22 | 39 | 307 | 42 | 127 | 35 | 180 | 49 |
| Girl-/boyfriend, not living together | 90 | 22 | 37 | 22 | 53 | 23 | 25 | 27 | 11 | 31 | 14 | 24 | 139 | 19 | 67 | 19 | 72 | 19 |
| Single | 143 | 36 | 74 | 43 | 69 | 30 | 34 | 37 | 13 | 38 | 21 | 37 | 285 | 39 | 165 | 46 | 120 | 32 |
| Educational level – highest finished |  |  |  |  |  |  |  |  |  |  |  |  |  |  |  |  |  |  |
| Compulsory school/high school | 336 | 76 | 153 | 80 | 183 | 73 | 82 | 85 | 34 | 97 | 48 | 77 | 638 | 82 | 329 | 88 | 309 | 77 |
| College/university | 88 | 24 | 27 | 20 | 61 | 27 | 15 | 15 | 1 | 3 | 14 | 23 | 137 | 18 | 44 | 12 | 93 | 23 |
| Occupation with demands of education at college/university level |  |  |  |  |  |  |  |  |  |  |  |  |  |  |  |  |  |  |
| Yes | 50 | 12 | 15 | 8 | 35 | 14 | 7 | 8 | 1 | 3 | 6 | 10 | 89 | 12 | 28 | 8 | 61 | 16 |
| No | 373 | 88 | 163 | 92 | 210 | 86 | 86 | 92 | 33 | 97 | 53 | 90 | 673 | 88 | 342 | 92 | 331 | 84 |
| Living area |  |  |  |  |  |  |  |  |  |  |  |  |  |  |  |  |  |  |
| City | 176 | 41 | 62 | 34 | 114 | 46 | 54 | 55 | 21 | 60 | 33 | 52 | 302 | 39 | 139 | 37 | 163 | 40 |
| Not city | 255 | 59 | 119 | 66 | 136 | 54 | 44 | 45 | 14 | 40 | 30 | 48 | 480 | 61 | 238 | 63 | 242 | 60 |
| Birth country |  |  |  |  |  |  |  |  |  |  |  |  |  |  |  |  |  |  |
| Sweden | 404 | 94 | 167 | 92 | 237 | 95 | 91 | 93 | 32 | 91 | 59 | 94 | 742 | 95 | 360 | 96 | 382 | 94 |
| Other | 27 | 6 | 14 | 8 | 13 | 5 | 7 | 7 | 3 | 9 | 4 | 6 | 40 | 5 | 17 | 4 | 23 | 6 |
| Smoking |  |  |  |  |  |  |  |  |  |  |  |  |  |  |  |  |  |  |
| No, not at all/seldom | 373 | 87 | 162 | 90 | 211 | 85 | 75 | 77 | 32 | 91 | 43 | 68 | 675 | 86 | 335 | 89 | 340 | 84 |
| Yes, daily/nearly daily | 57 | 13 | 19 | 10 | 38 | 15 | 23 | 23 | 3 | 9 | 20 | 32 | 107 | 13 | 42 | 11 | 65 | 16 |
| Body mass index |  |  |  |  |  |  |  |  |  |  |  |  |  |  |  |  |  |  |
| < 25 kg/m² | 293 | 71 | 119 | 66 | 174 | 75 | 66 | 70 | 21 | 62 | 45 | 75 | 524 | 70 | 232 | 64 | 292 | 77 |
| ≥ 25 kg/m² | 119 | 29 | 60 | 34 | 59 | 25 | 28 | 30 | 13 | 38 | 15 | 25 | 220 | 30 | 133 | 36 | 87 | 23 |
| Physical activity in leisure time |  |  |  |  |  |  |  |  |  |  |  |  |  |  |  |  |  |  |
| Moderate exercise-hard training | 354 | 83 | 147 | 82 | 207 | 84 | 82 | 84 | 29 | 83 | 53 | 84 | 652 | 85 | 306 | 84 | 341 | 86 |
| Sedentary | 72 | 17 | 32 | 18 | 40 | 16 | 16 | 16 | 6 | 17 | 10 | 16 | 117 | 15 | 61 | 16 | 56 | 14 |
| Chronic pain or ache ( > 3 months) |  |  |  |  |  |  |  |  |  |  |  |  |  |  |  |  |  |  |
| No | 333 | 78 | 153 | 86 | 180 | 72 | 62 | 64 | 25 | 71 | 37 | 60 | 593 | 76 | 309 | 83 | 284 | 70 |
| Yes | 94 | 22 | 25 | 14 | 69 | 28 | 35 | 36 | 10 | 29 | 25 | 40 | 185 | 24 | 64 | 17 | 121 | 30 |
| Symptoms of depression last month |  |  |  |  |  |  |  |  |  |  |  |  |  |  |  |  |  |  |
| No | 186 | 62 | 91 | 69 | 95 | 57 | 21 | 28 | 11 | 41 | 10 | 21 | 343 | 61 | 199 | 71 | 144 | 51 |
| Yes | 113 | 38 | 40 | 31 | 73 | 43 | 53 | 72 | 16 | 59 | 37 | 79 | 222 | 39 | 82 | 29 | 140 | 49 |
| Experienced health |  |  |  |  |  |  |  |  |  |  |  |  |  |  |  |  |  |  |
| Good or very good | 335 | 78 | 145 | 80 | 190 | 76 | 54 | 55 | 25 | 71 | 29 | 46 | 619 | 80 | 321 | 85 | 298 | 74 |
| Very bad, bad or moderately | 95 | 22 | 36 | 20 | 59 | 24 | 44 | 45 | 10 | 29 | 34 | 54 | 161 | 20 | 56 | 15 | 105 | 26 |
