# Supplementary material for: Changes in work situation and work ability in young female and male workers. A prospective cohort study
Source: BMC Public Health. 2012 Aug 24;12:694. doi: 10.1186/1471-2458-12-694 (PMC3508794; doi:10.1186/1471-2458-12-694)
Supplement: Additional file 2 — Table B. Descriptive characteristics of work factors at baseline and changes in work factors between baseline and 1- year follow-up for the study sample.(N = number of workers). Description of the data: this table gives a completion to the manuscript and show the exposure at baseline and the change in exposure between baseline – the 1-year follow-up, to better assess the results and the discussion in the article. [file 1471-2458-12-694-S2.doc]

| **Table B.** **Descriptive characteristics of work factors at baseline and changes in work factors between baseline and 1- year follow-up for the study sample.** **(N= number of workers)** | Baseline | | | | | | Plausible increased negative exposure, baseline – 1- year follow-up | | | | | | Plausible decreased negative exposure , baseline – 1- year follow-up | | | | | |
| --- | --- | --- | --- | --- | --- | --- | --- | --- | --- | --- | --- | --- | --- | --- | --- | --- | --- | --- |
|  | All workers  (N=1311) | | Males  (N=593, 45%) | | Females  (N=718, 55%) | | All workers | | Males | | Females | | All workers | | Males | | Females | |
| *Physical work factors* |  |  |  |  |  |  |  |  |  |  |  |  |  |  |  |  |  |  |
|  | N | % | N | % | N | % | N | % | N | % | N | % | N | % | N | % | N | % |
| Computer time, h/day, in general last month |  |  |  |  |  |  | 239 | 18 | 102 | 17 | 137 | 19 | 201 | 15 | 94 | 16 | 107 | 15 |
| < 2 h/day | 506 | 39 | 214 | 36 | 292 | 41 |  |  |  |  |  |  |  |  |  |  |  |  |
| 2-4 h/day | 368 | 28 | 192 | 33 | 176 | 25 |  |  |  |  |  |  |  |  |  |  |  |  |
| >4 h/day | 432 | 33 | 186 | 31 | 246 | 34 |  |  |  |  |  |  |  |  |  |  |  |  |
| Computer use 2 h with no breaks  > 10 min, last month |  |  |  |  |  |  | 378 | 29 | 158 | 27 | 220 | 31 | 364 | 28 | 169 | 28 | 195 | 27 |
| Never | 291 | 22 | 115 | 19 | 176 | 25 |  |  |  |  |  |  |  |  |  |  |  |  |
| Once in awhile | 369 | 28 | 146 | 25 | 223 | 31 |  |  |  |  |  |  |  |  |  |  |  |  |
| A couple of times per month | 198 | 15 | 90 | 15 | 108 | 15 |  |  |  |  |  |  |  |  |  |  |  |  |
| A couple of times per week | 202 | 16 | 109 | 18 | 93 | 13 |  |  |  |  |  |  |  |  |  |  |  |  |
| Most days | 248 | 19 | 133 | 23 | 115 | 16 |  |  |  |  |  |  |  |  |  |  |  |  |
| Hands above shoulder level last month |  |  |  |  |  |  | 246 | 19 | 108 | 18 | 138 | 19 | 273 | 21 | 129 | 22 | 144 | 20 |
| Never | 361 | 28 | 137 | 23 | 224 | 32 |  |  |  |  |  |  |  |  |  |  |  |  |
| < 1 h/day | 606 | 46 | 263 | 45 | 343 | 48 |  |  |  |  |  |  |  |  |  |  |  |  |
| 1-2 h/day | 190 | 15 | 112 | 19 | 78 | 11 |  |  |  |  |  |  |  |  |  |  |  |  |
| >2 h/day | 143 | 11 | 79 | 13 | 64 | 9 |  |  |  |  |  |  |  |  |  |  |  |  |
| Flexed or extended neck last month |  |  |  |  |  |  | 345 | 26 | 154 | 26 | 191 | 27 | 309 | 24 | 142 | 24 | 167 | 23 |
| Never | 238 | 18 | 84 | 14 | 154 | 22 |  |  |  |  |  |  |  |  |  |  |  |  |
| < 3 h/day | 546 | 42 | 286 | 49 | 260 | 36 |  |  |  |  |  |  |  |  |  |  |  |  |
| 3-5 h/day | 258 | 20 | 113 | 19 | 145 | 20 |  |  |  |  |  |  |  |  |  |  |  |  |
| >5 h/day | 261 | 20 | 106 | 18 | 155 | 22 |  |  |  |  |  |  |  |  |  |  |  |  |
| Flexed back last month |  |  |  |  |  |  | 297 | 23 | 152 | 24 | 155 | 22 | 293 | 22 | 140 | 24 | 153 | 21 |
| Never | 276 | 21 | 108 | 18 | 168 | 24 |  |  |  |  |  |  |  |  |  |  |  |  |
| < 0,5 h/day | 291 | 22 | 146 | 25 | 145 | 20 |  |  |  |  |  |  |  |  |  |  |  |  |
| 0,5-1 h/day | 272 | 21 | 137 | 23 | 135 | 19 |  |  |  |  |  |  |  |  |  |  |  |  |
| >1 h/day | 465 | 36 | 198 | 34 | 267 | 37 |  |  |  |  |  |  |  |  |  |  |  |  |
| Lifting 5-10 kg last month |  |  |  |  |  |  | 249 | 19 | 127 | 21 | 122 | 17 | 270 | 21 | 120 | 20 | 150 | 21 |
| 0-4 times/day | 518 | 42 | 169 | 31 | 349 | 52 |  |  |  |  |  |  |  |  |  |  |  |  |
| 5-15 times/day | 316 | 26 | 153 | 28 | 163 | 24 |  |  |  |  |  |  |  |  |  |  |  |  |
| 16-30 times/day | 147 | 12 | 85 | 15 | 62 | 9 |  |  |  |  |  |  |  |  |  |  |  |  |
| >30 times/day | 242 | 20 | 144 | 26 | 98 | 15 |  |  |  |  |  |  |  |  |  |  |  |  |
| Lifting 11-15 kg last month |  |  |  |  |  |  | 189 | 14 | 112 | 19 | 77 | 11 | 223 | 17 | 132 | 22 | 91 | 13 |
| 0-4 times/day | 685 | 59 | 242 | 45 | 443 | 70 |  |  |  |  |  |  |  |  |  |  |  |  |
| 5-15 times/day | 261 | 22 | 152 | 28 | 109 | 17 |  |  |  |  |  |  |  |  |  |  |  |  |
| 16-30 times/day | 105 | 9 | 63 | 12 | 42 | 7 |  |  |  |  |  |  |  |  |  |  |  |  |
| >30 times/day | 114 | 10 | 78 | 15 | 36 | 6 |  |  |  |  |  |  |  |  |  |  |  |  |
| Lifting 16-25 kg last month |  |  |  |  |  |  | 136 | 10 | 84 | 14 | 52 | 7 | 166 | 13 | 95 | 16 | 71 | 10 |
| 0-4 times/day | 809 | 71 | 314 | 59 | 495 | 82 |  |  |  |  |  |  |  |  |  |  |  |  |
| 5-15 times/day | 199 | 17 | 125 | 23 | 74 | 12 |  |  |  |  |  |  |  |  |  |  |  |  |
| 16-30 times/day | 57 | 5 | 36 | 7 | 21 | 3 |  |  |  |  |  |  |  |  |  |  |  |  |
| >30 times/day | 76 | 7 | 59 | 11 | 17 | 3 |  |  |  |  |  |  |  |  |  |  |  |  |
| Lifting > 25 kg last month |  |  |  |  |  |  | 115 | 9 | 70 | 12 | 45 | 6 | 131 | 10 | 81 | 14 | 50 | 7 |
| 0-4 times/day | 902 | 79 | 374 | 70 | 528 | 86 |  |  |  |  |  |  |  |  |  |  |  |  |
| 5-15 times/day | 157 | 14 | 99 | 19 | 58 | 10 |  |  |  |  |  |  |  |  |  |  |  |  |
| 16-30 times/day | 32 | 3 | 21 | 4 | 11 | 2 |  |  |  |  |  |  |  |  |  |  |  |  |
| >30 times/day | 53 | 4 | 39 | 7 | 14 | 2 |  |  |  |  |  |  |  |  |  |  |  |  |
| Forceful grip last month |  |  |  |  |  |  | 221 | 17 | 119 | 20 | 102 | 14 | 267 | 20 | 131 | 22 | 136 | 19 |
| Seldom or never | 707 | 54 | 227 | 39 | 480 | 67 |  |  |  |  |  |  |  |  |  |  |  |  |
| Several times/day | 397 | 31 | 220 | 37 | 177 | 25 |  |  |  |  |  |  |  |  |  |  |  |  |
| Several times/h | 152 | 12 | 108 | 18 | 44 | 6 |  |  |  |  |  |  |  |  |  |  |  |  |
| Several times/min | 44 | 3 | 33 | 6 | 11 | 2 |  |  |  |  |  |  |  |  |  |  |  |  |
| Use of vibrating tools |  |  |  |  |  |  | 58 | 4 | 33 | 6 | 25 | 3 | 46 | 4 | 31 | 5 | 15 | 2 |
| No | 965 | 78 | 318 | 58 | 647 | 93 |  |  |  |  |  |  |  |  |  |  |  |  |
| Yes, in work | 278 | 22 | 233 | 42 | 45 | 7 |  |  |  |  |  |  |  |  |  |  |  |  |
|  |  |  |  |  |  |  |  |  |  |  |  |  |  |  |  |  |  |  |
| *Psychosocial work factors* |  |  |  |  |  |  |  |  |  |  |  |  |  |  |  |  |  |  |
|  |  |  |  |  |  |  |  |  |  |  |  |  |  |  |  |  |  |  |
| Experiencing high job demands |  |  |  |  |  |  | 385 | 29 | 150 | 25 | 235 | 33 | 236 | 18 | 107 | 18 | 129 | 18 |
| Correspond very poorly | 79 | 6 | 34 | 6 | 45 | 6 |  |  |  |  |  |  |  |  |  |  |  |  |
| Correspond fairly poorly | 240 | 18 | 90 | 15 | 150 | 21 |  |  |  |  |  |  |  |  |  |  |  |  |
| Correspond fairly well | 612 | 47 | 285 | 49 | 327 | 46 |  |  |  |  |  |  |  |  |  |  |  |  |
| Correspond very well | 374 | 29 | 179 | 30 | 195 | 27 |  |  |  |  |  |  |  |  |  |  |  |  |
| Experiencing job control |  |  |  |  |  |  | 388 | 30 | 168 | 28 | 220 | 31 | 199 | 15 | 90 | 15 | 109 | 15 |
| Correspond very poorly | 16 | 1 | 8 | 1 | 8 | 1 |  |  |  |  |  |  |  |  |  |  |  |  |
| Correspond fairly poorly | 84 | 6 | 33 | 6 | 51 | 7 |  |  |  |  |  |  |  |  |  |  |  |  |
| Correspond fairly well | 689 | 53 | 309 | 52 | 380 | 53 |  |  |  |  |  |  |  |  |  |  |  |  |
| Correspond very well | 517 | 40 | 239 | 41 | 278 | 39 |  |  |  |  |  |  |  |  |  |  |  |  |
| Experiencing social support at work |  |  |  |  |  |  | 420 | 32 | 179 | 30 | 241 | 34 | 239 | 18 | 103 | 17 | 136 | 19 |
| Correspond very poorly | 45 | 3 | 19 | 3 | 26 | 4 |  |  |  |  |  |  |  |  |  |  |  |  |
| Correspond fairly poorly | 176 | 14 | 81 | 14 | 95 | 13 |  |  |  |  |  |  |  |  |  |  |  |  |
| Correspond fairly well | 556 | 43 | 260 | 44 | 296 | 41 |  |  |  |  |  |  |  |  |  |  |  |  |
| Correspond very well | 527 | 40 | 227 | 39 | 300 | 42 |  |  |  |  |  |  |  |  |  |  |  |  |
| Experiencing reward relative to effort |  |  |  |  |  |  | 415 | 32 | 189 | 32 | 226 | 31 | 326 | 25 | 133 | 22 | 193 | 27 |
| Correspond very poorly | 124 | 10 | 57 | 10 | 67 | 9 |  |  |  |  |  |  |  |  |  |  |  |  |
| Correspond fairly poorly | 310 | 24 | 131 | 22 | 179 | 25 |  |  |  |  |  |  |  |  |  |  |  |  |
| Correspond fairly well | 582 | 44 | 263 | 45 | 319 | 45 |  |  |  |  |  |  |  |  |  |  |  |  |
| Correspond very well | 287 | 22 | 136 | 23 | 151 | 21 |  |  |  |  |  |  |  |  |  |  |  |  |
| Experiencing negative influence of job demands on private life |  |  |  |  |  |  | 411 | 31 | 172 | 29 | 239 | 33 | 349 | 27 | 149 | 25 | 200 | 28 |
| Very seldom | 552 | 42 | 275 | 47 | 277 | 39 |  |  |  |  |  |  |  |  |  |  |  |  |
| Fairly seldom | 237 | 18 | 108 | 18 | 129 | 18 |  |  |  |  |  |  |  |  |  |  |  |  |
| Sometimes | 346 | 27 | 139 | 23 | 207 | 29 |  |  |  |  |  |  |  |  |  |  |  |  |
| Fairly often | 116 | 9 | 47 | 8 | 69 | 10 |  |  |  |  |  |  |  |  |  |  |  |  |
| Very often | 55 | 4 | 22 | 4 | 33 | 4 |  |  |  |  |  |  |  |  |  |  |  |  |
| Work outside the workplace last month |  |  |  |  |  |  | 317 | 24 | 138 | 23 | 179 | 25 | 209 | 16 | 95 | 16 | 114 | 16 |
| Never | 817 | 63 | 380 | 65 | 437 | 61 |  |  |  |  |  |  |  |  |  |  |  |  |
| Once in awhile | 267 | 20 | 107 | 18 | 160 | 23 |  |  |  |  |  |  |  |  |  |  |  |  |
| A couple of times per month | 107 | 8 | 47 | 8 | 60 | 8 |  |  |  |  |  |  |  |  |  |  |  |  |
| A couple of times per week | 72 | 6 | 36 | 6 | 36 | 5 |  |  |  |  |  |  |  |  |  |  |  |  |
| On the whole every day | 40 | 3 | 20 | 3 | 20 | 3 |  |  |  |  |  |  |  |  |  |  |  |  |
| Overtime work (>12 h/day) last month |  |  |  |  |  |  | 319 | 24 | 167 | 28 | 152 | 21 | 264 | 20 | 123 | 21 | 141 | 20 |
| 0 times | 739 | 57 | 299 | 51 | 440 | 61 |  |  |  |  |  |  |  |  |  |  |  |  |
| 1-2 times | 316 | 24 | 144 | 24 | 172 | 24 |  |  |  |  |  |  |  |  |  |  |  |  |
| 3-8 times | 169 | 13 | 90 | 15 | 79 | 11 |  |  |  |  |  |  |  |  |  |  |  |  |
| 9-15 times | 44 | 3 | 26 | 4 | 18 | 3 |  |  |  |  |  |  |  |  |  |  |  |  |
| >15 times | 42 | 3 | 34 | 6 | 8 | 1 |  |  |  |  |  |  |  |  |  |  |  |  |
| Need to be reachable by mobile phone in duty after working hours last month. |  |  |  |  |  |  | 340 | 26 | 172 | 29 | 168 | 23 | 249 | 19 | 117 | 20 | 132 | 18 |
| Never | 685 | 53 | 293 | 50 | 392 | 55 |  |  |  |  |  |  |  |  |  |  |  |  |
| Once in awhile | 258 | 20 | 104 | 17 | 154 | 22 |  |  |  |  |  |  |  |  |  |  |  |  |
| A couple of times per month | 113 | 9 | 65 | 11 | 48 | 7 |  |  |  |  |  |  |  |  |  |  |  |  |
| A couple of times per week | 71 | 5 | 34 | 6 | 37 | 5 |  |  |  |  |  |  |  |  |  |  |  |  |
| On the whole every day | 176 | 13 | 94 | 16 | 82 | 11 |  |  |  |  |  |  |  |  |  |  |  |  |
| Noise annoyance at the workplace last month |  |  |  |  |  |  | 427 | 33 | 190 | 32 | 237 | 33 | 329 | 25 | 147 | 25 | 182 | 25 |
| Never | 174 | 13 | 98 | 17 | 76 | 11 |  |  |  |  |  |  |  |  |  |  |  |  |
| Once in awhile | 261 | 20 | 103 | 17 | 158 | 22 |  |  |  |  |  |  |  |  |  |  |  |  |
| A couple of times per month | 163 | 13 | 67 | 11 | 96 | 13 |  |  |  |  |  |  |  |  |  |  |  |  |
| A couple of times per week | 195 | 15 | 81 | 14 | 114 | 16 |  |  |  |  |  |  |  |  |  |  |  |  |
| On the whole every day | 512 | 39 | 239 | 41 | 273 | 38 |  |  |  |  |  |  |  |  |  |  |  |  |
